# Draft Genome Sequence of *Taylorella equigenitalis* Strain MCE529, Isolated from a Belgian Warmblood Horse

**DOI:** 10.1128/genomeA.01214-14

**Published:** 2014-11-26

**Authors:** Laurent Hébert, Fabrice Touzain, Claire de Boisséson, Marie-France Breuil, Fabien Duquesne, Claire Laugier, Yannick Blanchard, Sandrine Petry

**Affiliations:** a ANSES, Dozulé Laboratory for Equine Diseases, Bacteriology and Parasitology Unit, Goustranville, France; b ANSES, Ploufragan/Plouzané Laboratory, Viral Genetics and Bio-Security Unit, Université Européenne de Bretagne, Ploufragan, France; c ANSES, Dozulé Laboratory for Equine Diseases, Goustranville, France

## Abstract

*Taylorella equigenitalis* is the causative agent of contagious equine metritis (CEM), a sexually transmitted infection of horses. We herein report the genome sequence of *T. equigenitalis* strain MCE529, isolated in 2009 from the urethral fossa of a 15-year-old Belgian Warmblood horse in France.

## GENOME ANNOUNCEMENT

*Taylorella equigenitalis* is a slow-growing capnophilic Gram-negative coccobacillus, classified in the *Burkholderiales* order and the *Alcaligenaceae* family (1). It is the etiological agent of contagious equine metritis (CEM), a highly contagious sexually transmitted infection of horses characterized in infected mares by abundant mucopurulent vaginal discharge and a variable degree of vaginitis, endometritis, and cervicitis. CEM usually results in temporary infertility or early abortion (2). The presence of *T. equigenitalis* in stallions does not cause clinical signs and long-term asymptomatic carrier mares have also been reported (3). CEM is a World Organisation for Animal Health (OIE) notifiable disease, and is considered as one of the most regulated equine diseases worldwide (4). The recent development of a multilocus sequence typing (MLST) scheme for taylorellae (5) offered a comprehensive overview of the genetic diversity of taylorellae. To date, the genome sequences of only three *T. equigenitalis* strains have been reported (6, 7) and the genome sequences of numerous *T. equigenitalis* sequence types (STs) remain to be characterized.

We herein report the genome sequence of *T. equigenitalis* MCE529, which was isolated in 2009 from the urethral fossa of a 15 year old asymptomatic carrier, Belgian Warmblood stallion from a stud farm in Lower Normandy (France). Sequence typing of this strain using the MLST database *Taylorella* (http://pubmlst.org/taylorella/), revealed its membership in the previously non sequenced ST16 of the clonal complex 2 (5).

The genome of *T. equigenitalis* strain MCE529 was sequenced by Ion Torrent technology (Life Technologies). The library was constructed using the Ion Xpress Plus fragment library kit, with size selection by electrophoresis. Emulsion PCR was performed with an Ion OneTouch 2 system followed by enrichment with an Ion OneTouch ES system, both using the Ion PI template OT2 200 kit v3 (Life Technologies). Sequencing was run on the Ion Torrent Proton (Life Technologies) loaded with a P1 chip as described in the manufacturer’s protocol. In total, 2.54 million reads (mean length, 181 bases) generated 422 Mb of data, of which 1,087,540 reads were assembled (estimated coverage <80×) using MIRA version 4.0rc1 (8) into 26 large contigs (>500 bp), giving a consensus length of 1,668,004 bp. The k-mer size used by MIRA was deduced by using kmergenie version 1.5658 (9). Mauve 2.3.1 (10) was used to order the contigs and compare the genome with that of *T. equigenitalis* strain MCE9 (accession no. CP002456). Annotation was added by the NCBI Prokaryotic Genome Annotation Pipeline, released 2013 (http://www.ncbi.nlm.nih.gov/genome/annotation_prok/), yielding 1,564 open reading frames (ORFs) and 36 tRNAs. The average G+C content of the draft genome sequences is 37.4%. One clustered regularly interspaced short palindromic repeat (CRISPR)/Cas loci and one restriction/modification system were detected.

### Nucleotide sequence accession numbers.

This whole-genome shotgun project has been deposited at DDBJ/EMBL/GenBank under the accession no. JRMO00000000. The version described in this paper is the first version JRMO01000000.

## References

[1] Sugimoto C Isayama Y Sakazaki R Kuramochi S 1983 Transfer of *Haemophilus equigenitalis* Taylor et al. 1978 to the genus *Taylorella* gen. nov. as *Taylorella equigenitalis* comb. nov. Curr. Microbiol. 9:155–162. 10.1007/BF01567289.

[2] Nakashiro H Naruse M Sugimoto C Isayama Y Kuniyasu C 1981 Isolation of *Haemophilus equigenitalis* from an aborted equine fetus. Natl. Inst. Anim. Health Q. (Tokyo) 21:184–185. 7341994

[3] Matsuda M Moore JE 2003 Recent advances in molecular epidemiology and detection of *Taylorella equigenitalis* associated with contagious equine metritis (CEM). Vet. Microbiol. 97:111–122. 10.1016/j.vetmic.2003.08.001. 14637043

[4] Schulman ML May CE Keys B Guthrie AJ 2013 Contagious equine metritis: artificial reproduction changes the epidemiologic paradigm. Vet. Microbiol. 167:2–8. 10.1016/j.vetmic.2012.12.021. 23332460

[5] Duquesne F Hébert L Breuil MF Matsuda M Laugier C Petry S 2013 Development of a single multi-locus sequence typing scheme for *Taylorella equigenitalis* and *Taylorella asinigenitalis*. Vet. Microbiol. 167:609–618. 10.1016/j.vetmic.2013.09.016. 24139720

[6] Hauser H Richter DC van Tonder A Clark L Preston A 2012 Comparative genomic analyses of the Taylorellae. Vet. Microbiol. 159:195–203. 10.1016/j.vetmic.2012.03.041. 22541164

[7] Hébert L Moumen B Duquesne F Breuil M-F Laugier C Batto J-M Renault P Petry S 2011 Genome sequence of *Taylorella equigenitalis* MCE9, the causative agent of contagious equine metritis. J. Bacteriol. 193:1785. 10.1128/JB.01547-10. 21278298 PMC3067654

[8] Chevreux B Wetter T Suhai S 1999 Genome sequence assembly using trace signals and additional sequence information, p 45–56 *In* Giegerich R (ed), Proceedings of the German Conference on Bioinformatics. GBF-Braunschweig and University of Bielefeld, Bielefeld, Germany.

[9] Chikhi R Medvedev P 2014 Informed and automated k-mer size selection for genome assembly. Bioinformatics 30:31–37. 10.1093/bioinformatics/btt310. 23732276

[10] Rissman AI Mau B Biehl BS Darling AE Glasner JD Perna NT 2009 Reordering contigs of draft genomes using the Mauve aligner. Bioinformatics 25:2071–2073. 10.1093/bioinformatics/btp356. 19515959 PMC2723005

